# Pediatric Hypothermic Submersion Injury and Protective Factors Associated with Optimal Outcome: A Case Report and Literature Review

**DOI:** 10.3390/children5010004

**Published:** 2017-12-27

**Authors:** Daniel Kriz, Juan Piantino, Devin Fields, Cydni Williams

**Affiliations:** 1 Department of Pediatrics, Division of Psychology, Neuro-Critical Care Program, Oregon Health and Science University, Portland, OR 97239, USA; fiel4927@pacificu.edu; 2 Programs for Evaluation, Development and Learning, St. Charles Healthcare Systems, Bend, OR 97701, USA; 3 Department of Pediatrics, Division Pediatric Neurology, Neuro-Critical Care Program, Oregon Health and Science University, Portland, OR 97239, USA; piantino@ohsu.edu; 4 Department of Pediatrics, Division Pediatric Critical Care, Neuro-Critical Care Program, Oregon Health and Science University, Portland, OR 97239, USA; willicyd@ohsu.edu

**Keywords:** drowning, hypothermia, out-of-hospital cardiac arrest, neuropsychological tests, outcome assessment, critical care outcome, pediatric

## Abstract

Drowning is the 3rd leading cause of unintentional injury death worldwide, with the highest rates of fatality among young children. Submersion injuries with cardiac arrest can lead to long-term neurologic morbidity. Severe hypothermic submersion injuries have complex treatment courses and survivors have variable neurocognitive outcomes. We describe the course of a hypothermic submersion injury in a 6-year-old previously healthy boy. The description includes premorbid and post-injury neurocognitive functioning. A review of the literature of pediatric cold-water submersion injury was performed. Despite prolonged cardiopulmonary resuscitation (>100 min) and water temperature well above freezing, our patient had an optimal neurocognitive outcome following hypothermic submersion injury. Available literature is limited but suggests that increased submersion time, increased duration of resuscitation, and higher water temperatures are associated with worse outcomes. Care guidelines have been created, but outcomes related to these guidelines have not been studied. Our case highlights potential important determinants of outcome after drowning. Incident specific characteristics and therapeutic interventions should be considered when evaluating this population. Treatment guidelines based on currently available literature may fail to incorporate all potential variables, and consideration should be given to prolonged resuscitative efforts based on individual case characteristics until further data is available.

## 1. Introduction

Drowning is the 3rd leading cause of unintentional injury death worldwide, with the highest drowning rates among children [1]. In a recent global childhood unintentional injury surveillance project, drowning, or submersion injury, had the highest mean injury severity score [2]. Pediatric submersion injury with cardiac arrest is associated with high morbidity and in-hospital mortality, though case reports have reported good outcomes when associated with cold-water submersion and severe hypothermia. Severe hypothermia is defined as a core temperature of <28 °C.

Semple-Hess and Campwala recently reviewed pediatric submersion injuries with regard to emergency care and resuscitation and published clinical pathways for the management of hypothermic submersion injury in children [3]. To our knowledge, no studies have determined whether adherence to these pathways is associated with improved outcomes.

The majority of long-term sequelae in submersion injuries are neurocognitive, but standardized outcome measures are frequently not used in clinical evaluation. Permanent neurologic damage is not uncommon and outcome is determined by the duration and severity of the hypoxic-ischemic insult [4], though factors involving submersion injuries mitigating the insult are not fully elucidated. There are many multi-center studies involving post-cardiac arrest syndrome and outcomes that warrant reading, but the studies are not specific to submersion injuries and are therefore not within the scope of this paper [5]. No multicenter studies addressing factors involving submersion injuries that may predict neurological outcomes have been performed [3]. The available literature consists mostly of case reports and single institution studies [3]. To date, findings and predictors of good neurological outcomes in pediatric drowning are not well understood. Historical retrospective studies have indicated variable outcomes in those with anoxic injury from multiple mechanisms, including avalanche, cold weather, and cold-water submersion [6,7,8,9]. Studies have not fully evaluated the factors or combination of factors relating to incident characteristics and therapies resulting in optimal outcomes. Specific to submersion injuries, water temperature, duration of submersion at various temperatures, core temperature at retrieval, body pH, potassium levels, quality of resuscitation, in-hospital management, and rewarming rate may all play a role [3,4,10,11,12,13].

Here we describe a premorbid academically gifted six-year-old boy and his treatment course as it relates to a cold water submersion injury with severe hypothermia, prolonged cardiopulmonary resuscitation, and hypoxic respiratory failure. He was discharged with intact neurocognitive status and returned to academic giftedness at follow-up. We hypothesized that our patient’s optimal outcome was related to a multitude of unique incident characteristics in addition to therapeutic interventions. We aimed to review the literature to determine factors associated with improved neurocognitive outcomes following pediatric hypothermic submersion injuries and report other factors that should be considered when caring for these patients.

## 2. Materials and Methods

The case-specific information was obtained via retrospective chart review. Institutional Review Board (IRB) was consulted and they deemed this project as not having Protected Health Information (PHI), so IRB approval was not necessary. A review of the available literature was performed by querying Pubmed and Ovid databases using the terms “near drowning/drowning and hypothermia”, “child or adolescent”, “anoxia”, “cold water submersion”, “outcomes”, and various combinations of the above within the last 10 years. Five retrospective, single center studies were identified. Other studies were identified, but hypothermia samples were not related to cold water submersion (i.e., avalanche victims), or the cold water submersion sample within the larger sample was not parsed out for separate analysis, so they are not reported here [14,15,16]. Patient information was obtained via retrospective chart review. The methods are consistent with Case Report (CARE) guidelines.

## 3. Results

### 3.1. Patient Description

A previously healthy six-year-old Caucasian male presented to the Pediatric Intensive Care Unit (PICU) via transport following a submersion injury and prolonged resuscitation in a rural area Emergency Room (ER). The event took place in mid-summer with outside temperature approximately 31 °C. The patient was submerged in water at 55 °F/12.77 °C [12] for an estimated 30 min prior to Coast Guard arrival. He was found in a life jacket under a capsized boat after the boat struck some rocks during a river fishing trip and was initially conscious within an air pocket under the vessel. During rescue efforts, he was unwitnessed for approximately 10 min while other victims were rescued and was subsequently found submerged and asystolic. He was removed from the water and cardiopulmonary resuscitation (CPR) began. He was transported to a local ER via ground transport where he was unresponsive, pupils fixed and dilated, with CPR in progress. He arrived at the ER at approximately 1600. He received one defibrillation on ER arrival per the transport team’s automated external defibrillator prior to rhythm evaluation by ER team. The first obtainable body temperature in the ER was 28.8 °C, and rhythm was bradycardic at 20 beats per minute (bpm). He showed an initial pH of 6.5, arterial partial pressure of carbon dioxide (PaCO2) level of 26 mmHg, an Na level of 150, a K level of 2.6, and a lactate level of 13. After 60 min of CPR with rhythms of asystole, bradycardia, and pulseless electrical activity, agonal breathing was noted and the PICU was consulted. The PICU recommended active rewarming with warmed oxygen and intravenous fluids, external bear hugger warming, epinephrine infusion, and bicarbonate administration. Bradycardia with inadequate perfusion was persistent, despite high doses of epinephrine at 0.5 mg/kg/min, requiring ongoing CPR for an additional 42 min (total CPR: 102 min) until the ER physician stopped CPR in anticipation of patient death. Given the rural setting and long distance to a tertiary center with extracorporeal services, no further therapies were available. At the cessation of CPR, the patient was noted to have a heart rate of 54 with a blood pressure of 79/37. Resuscitation was continued and he was stabilized for ground transport over the next 2 h, requiring high ventilator settings for large volume pulmonary edema and acute respiratory distress syndrome (ARDS), ongoing fluid resuscitation, and epinephrine infusion. During transport approximately 4 h after the event, he was noted to have reactive and equal pupils, a temperature of 34.4 °C, and spontaneous respirations and was weaned off epinephrine infusion to maintain systolic blood pressure (80–90). He continued to receive active rewarming with warmed oxygen, intravenous fluids, and external bear hugger warming. Purposeful extremity movements requiring administration of fentanyl and versed for sedation were also noted during transport. He was transported with head of bed (HOB) elevated to 30 degrees per standard for intubated patients; end tidal CO_2_ maintained at 30–40, and oxygen saturations maintained above 92%. No other significant events occurred during transport.

Upon admission to the PICU approximately 7 h after the event, his temperature was normalized to 37.7 °C (an estimated warming rate of 1.2 °C/h). He required ongoing fluid resuscitation but did not require re-initiation of vasopressors. He had significant hypoxia with ARDS requiring high pressures and high levels of inspired oxygen. Initial neurologic exam noted purposeful movements with spontaneous eye opening, but sedative infusions were required for management of hypoxic respiratory failure precluding detailed examination. Head computed tomography was conducted approximately 9 h after admission when the patient’s respiratory status stabilized and was negative for acute injury with preserved gray white differentiation. Electroencephalogram was started 12 h after admission and showed posterior slowing with diffuse beta waves, but no seizures. No other traumatic injuries were identified. Sedative infusions and ventilator settings were weaned over the next 3 days as lung disease improved, revealing an intact neurologic exam. Further head imaging with magnetic resonance imaging was planned for 3–5 days after arrest but was deferred given that a normal neurologic exam on Pediatric Neurology evaluation and findings would not have influenced medical management at that time.

At 5 days post-injury, neuropsychology was consulted to assess parental stress and provide a patient Mental Status Exam (MSE). Premorbid cognitive status was superior, substantiated by school records (Table 1) qualifying for the Gifted and Talented Program. The PICU MSE indicated auditory and written perseveration, sequencing challenges, and notable confabulation. Working Memory was grossly intact. Possible receptive language phonological challenges were noted. Speech was difficult to understand due to softness and whispering (presumed secondary to intubation inflammation). Expressive language was disorganized, speech was dysfluent yet he coordinated verbal communication with eye contact and gestures. He made simple calculation errors. Amnesia of the event was reported. Declarative and procedural memory was grossly intact. Fine and gross motor skills were grossly intact. Attention/concentration was adequate. Behavioral/emotional status was indicative for apprehensiveness to talk with examiner out of fear of physical exam.

At 10 days post-injury, neuropsychology conducted an MSE and a brief standardized evaluation (NIH Toolbox and Wide Range Assessment of Memory and Learning, 2nd edition; Table 2). MSE was normal with the exception of a mild slow processing speed and mild speech dysfluency. Standardized testing resulted in superior vocabulary and sequencing memory, average delayed verbal recall memory, and intact executive functioning with the exception of borderline impaired processing speed. Behavioral and emotional health was within normal limits.

At one month post-injury, the patient was evaluated in the outpatient neurocritical care follow-up clinic with a pediatric neurologist, indicating no residual neurological deficits, but with reported mild fears associated with natural disasters.

At six months post-injury, the patient was again seen in the outpatient neurocritical care follow-up clinic with a pediatric neurologist and a pediatric neuropsychologist. No neurologic deficits were noted and his fears had resolved. Serial standardized neuropsychological testing (Table 1) indicated intact abilities with the exception of low average processing speed, which was an increase from scores obtained at discharge. Variability in scores was interpreted to be due to mild impulsivity noted at the 6 month follow-up. Results from the Behavior Assessment System for Children, Second Edition, Parent-Response Form indicated no at-risk or clinical elevations. Recent Academic Achievement scores were commensurate with premorbid functioning. He was subsequently discharged from the neurocritical care follow-up program.

### 3.2. Literature Review

We reviewed recent literature within the last 10 years identifying three retrospective studies looking at pediatric neurocognitive outcomes associated with hypothermia and cardiac arrest. None of the studies were multi-site, and they all occurred prior to the publication of the Pediatric Emergency Medicine Practice clinical pathway. Other studies were identified but included other means of obtaining hypothermia (i.e., avalanche and cold weather), and the submersion injury sample within the larger sample was not parsed out for separate analysis, so those studies are not reported here [14,15,16]. The reviewed studies showed that a resuscitation time <30 min, early CPR, the female gender, bradycardia, and slow rewarming speed were associated with improved outcomes. As these are retrospective studies, all possible variables were not evaluated or reported in each publication and outcome measures were dissimilar. As a way to demonstrate how many of the recent studies measured outcomes, a brief description of these studies is given in Table 3, and implications are addressed in the discussion.

## 4. Discussion

Severe hypothermic submersion injuries have complex treatment courses with variable survival rates and neurocognitive outcomes. Historical case studies of pediatric patients following hypothermic cardiac arrest with minimal or no neurologic sequelae has led to treatment recommendations and protocols using advanced therapies, such as extracorporeal support, even in cases of prolonged resuscitation. Other reports have led to guidelines that limit resuscitation efforts based on duration of CPR, submersion times, or water temperature. Intact neurological functioning after sustaining such an event is likely associated with a combination of variables ranging from incident characteristics to treatment course. Submersion times, water temperature, immediate resuscitation, quality and duration of resuscitation, idioventricular bradycardia, rewarming speed, and gender have been associated with improved outcomes in prior reports [12,13,18,22].

In our case, prolonged duration in the water of 30 min prior to full submersion and the cardiac arrest likely allowed for cooling of the patient’s core temperature prior to cardiac arrest and may play a key role in his optimal outcome despite water temperatures well above freezing. Literature has cited prolonged submersion as a risk factor for poor outcome [10], but duration in the water prior to submersion has not been evaluated. Additionally, water temperatures <5 °C have been associated with good outcomes, but time in warmer water prior to arrest has not been evaluated. We hypothesize our patient received protective effects in warmer water given time to equilibrate toward the water temperature prior to arrest. Therapeutic guidelines limiting intervention based solely on water temperature or submersion time may not fully encompass patients with the potential for good outcomes and should consider duration in the water prior to full submersion and arrest.

Immediate and quality resuscitation is an important factor in improving the possibility of optimal outcomes [17]. Our patient’s unwitnessed period was short, likely indicating relatively quick resuscitation efforts after arrest by trained personnel providing optimal CPR as compared to bystander CPR. Bradycardia was the predominant rhythm documented during the resuscitation, possibly indicates some native cardiac output, and may contribute to improved outcomes in pediatric cardiac arrest [19]. Prolonged resuscitation has been associated with poor outcomes, and one recent study’s findings caused the author to question the therapeutic value of prolonged resuscitation in pediatric submersion injury with cardiac arrest and hypothermia [17]. However, without more robust research providing a better understanding of the incident and therapeutic factors associated with outcomes, case reports such as ours oppose guidelines that limit resuscitation efforts in pediatric hypothermic submersion injuries based on resuscitation time.

Our patient’s initial core temperature was severely hypothermic at 28.8 °C and he was slowly rewarmed (estimated rate of 1.2 °C/h) to normothermia over several hours with limited passive and active techniques given the rural setting. Slow rewarming has been hypothesized to improve outcomes in extracorporeal rewarming [13] but may also be important when those therapies are not available, as in our case. Our patient significantly improved once his core temperature reached 34 °C. Guidelines suggest a target of 32 °C prior to considering cessation of resuscitation [23], but there may be other mitigating factors, and consideration should be given to higher temperature thresholds in certain cases. For example, seeing increasing evidence of neurologic function with rewarming, predominant bradycardic rhythm during resuscitation, and incident characteristics should be considered. The optimal rate of rewarming is unknown, but limiting the rate of rewarming can decrease complications [13,24] and may improve outcomes. Even without access to extracorporeal therapies, rewarming methods can be successful, and continuation of resuscitation to achieve higher temperatures should likely be considered in certain cases.

Our case was compared with studies published in the last 10 years (Table 3), where outcomes with cold water submersion hypothermic cardiac arrest in pediatric non-fatal drownings were analyzed to determine if there were variables that we did not consider in our optimal patient outcome and to identify what measures were used to determine neurocognitive outcomes. This comparison yielded that our study had more specific measures used to assess neurocognitive outcomes. All of the variables within these studies that were attributed to optimal outcome were considered in our case report, and many of the studies did not include important variables in their analysis. None of the studies considered the duration of water emersion prior to submersion as a variable when looking at outcomes.

Since the Clinical Pathway for the Severely Hypothermic Pediatric Drowning Victims guidelines were published in 2014 [3], no case reports have been published as a result of following those guidelines. The optimal outcome of our patient coincided with treatment consistent with the guidelines, but a combination of other factors may also be associated. Our patient received early and sustained CPR with epinephrine every 4–20 min (increasing interval with increasing code duration and start of epinephrine infusion), defibrillation, and slow passive and active rewarming as per the guidelines. His hemodynamic stability and neurologic exam notably improved with increasing temperatures, starting with the appearance of spontaneous breaths and progressing to pupillary reactivity with subsequent purposeful movements once temperatures were documented above 34 °C. This patient’s resuscitation was prolonged, partly due to the limited resources available in the rural ER to aid rewarming and the distance to tertiary care facilities precluding early transfer. Despite this prolonged duration of CPR including nearly stopping resuscitative efforts, which is considered in the guidelines, our patient had a remarkable recovery to his premorbid status with no deficits identified on detailed neuropsychological evaluation at 6 month follow-up.

Prior studies have not used standardized outcome measures of neurocognitive function. In fact, many reports have focused on mortality or gross measures of functional status without detailed neuropsychological evaluation. However, some studies have highlighted the need for these evaluations in submersion victims and in the general critical care population to identify important morbidities after discharge [19,25]. These detailed evaluations are more likely to identify deficits than gross measures of neurologic function and may identify important morbidities affecting patients over the long term and requiring intervention. There is a need to standardize outcome measures in these patients to advance future studies and understanding of this population, but also to provide patients with comprehensive follow-up care and to provide precise rehabilitative treatment plans.

## 5. Conclusions

Many pediatric hypothermic cardiac arrest studies incorporate various routes to hypothermia (i.e., avalanche, cold weather, and non-fatal drownings) and utilize a variety of outcome measures. Given incident specific variables that may impact neurocognitive outcomes in cold-water submersion injury, such as water temperature and length of submersion, studies evaluating outcomes in a variety of injury mechanisms may mask some important factors contributing to improved outcomes. Incident specific characteristics, therapeutic interventions, and combinations of the aforementioned should all be considered when looking at prognosis in this population. Despite prolonged cardiopulmonary resuscitation (>100 min) and water temperature well above freezing, our patient had an optimal neurocognitive outcome following hypothermic submersion injury. Our patient’s optimal outcome was likely due to a variety of contributing factors. The short interval from rescue to CPR by trained personnel, predominant bradycardic rhythm, and slow rewarming were key contributors. However, we hypothesize that our patient’s prolonged duration in the water of 30 min prior to full submersion and cardiac arrest was also a key contributing factor. This likely allowed cooling of the patient’s core temperature prior to cardiac arrest and may play a key role in his optimal outcome despite water temperatures well above freezing. Treatment guidelines based on currently available literature may fail to incorporate all potential variables, and consideration should be given to continued resuscitative efforts based on individual case characteristics until further data is available. Future research and case reports should incorporate Utstein-style guidelines for drowning-related resuscitation, as the documentation thereof includes specific information on incident characteristics, emergency response, treatment course, and neurological outcome. Neuropsychological evaluation is needed to identify sequelae that may be missed on gross neurologic evaluations to provide comprehensive outcomes research and optimal patient care.

## Figures and Tables

**Table 1:** Case study school performance pre- and post-morbid.

Skill	Pre-Morbid Percentile	6 Month Follow-Up Percentile
Word Reading Fluency	99%	92%
Passage Reading Fluency	Not assessed	98%
Common Core Stat Standard Mathematical Understanding	97%	95%

**Table 2:** Case Study Neuropsychological Assessment Results.

Cognition Area	Baseline at Discharge Age Corrected Standard Score	6 Month Follow-Up Corrected Standard Score
NIH Toolbox Picture Vocabulary	125.3	116
NIH Toolbox Pattern Comparison Processing Speed	70	83
NIH Toolbox Picture Sequence Memory	126	104
NIH Toolbox Flanker Inhibitory Control and Attention	111	113
WRAML2 List Learning Immediate Recall	115	110
WRAML2 List Learning Delayed Recall	100	105
WASI-II Vocabulary	NA	129
WASI-II Matrix Reasoning	NA	112
WRAT-4 Word Reading	NA	135
WRAT-4 Spelling	NA	129
WRAT-4 Math Computation	NA	126
WISC-V Coding	NA	115
Grooved Pegboard Dominant Hand (R)	NA	99
Grooved Pegboard Non-Dominant Hand	NA	108

NIH Toolbox iPad version 10.10.

**Table 3:** Published studies since 2007 analyzing outcomes with cold water submersion hypothermic cardiac arrest in pediatric non-fatal drownings.

Citation	N	Variables Considered in Analysis		Notable Variables Not Considered in Analysis	Measurement of Outcomes
Kieboom et al. [17]	N = 160			-Duration of water emersion prior to submersion -Water temperature -Method of assessing neurological function/outcome -Premorbid functioning	-In group that needed resuscitation for >30 min: -89% died. -11% survived in vegetative state or with severe cognitive damage using the Pediatric Cerebral Performance Scale (PCPC).
		-Age -Body of water	-Gender -Season		
		-Duration of CPR	-Body temp		
		-Initial blood gases	-pH, BE		
		-ECLS	-Initial cardiac rhythm		
		-Duration of submersion	-Life support at scene		
		-Neurological outcome			
Coskun et al. [18]	N = 5	-Age	-Gender	-Duration of water emersion prior to submersion -Water temperature -Length of resuscitation -Method of assessing neurological function/outcome -Premorbid functioning	Of the survivors and after a prolonged hospital stay: Children were assessed at discharge using the PCPC: -1 child was without any neurological injury. -1 child had mild neurological deficit. -3 children had severe neurological deficit. At 10–15 months follow-up: -2 children died of pulmonary complications. -1 child lost to follow-up. -2 were without neurological deficit (PCPC).
		-BMI	-Submersion time		
		-Asystole	-Body temp.		
		-pH	-Base excess		
		-Potassium	-Blood Glucose		
		-ECC duration	-ECMO		
		-Rewarming speed	-Ventricular bradycardia		
		-Ventricular fib	-Thoracic cannulation		
		-Femoral/iliac cannulation			
Eich et al. [19]	N = 12	Utstein-Style Guidelines for Drowning-Related Resuscitation:		-Duration of water emersion prior to submersion	Out of 12 children (22 months to 7.5 years of age): -5 survived to hospital discharge. -7 died in hospital. -2 survivors recovered fully. -3 remained in a vegetative state. 2 fully recovered patients were female measured by the PCPC: -low serum K+ concentrations -slow rewarming speed -received early basic life support -showed idioventricularbradycardia -cannulated via the iliac vessels; one switched to direct cardiac cannulation via median sternotomy.
		-Age	-Gender		
		-Accident details	-Ethnicity/race		
		-Pre-morbid health	-Residence		
		-Body of water	-Water temp.		
		-EMS called	-Submersion time		
		-Rescue time	-CPR method		
		-CPR quality	-EMS vehicle		
		-Initial Vitals	-GCS at rescue		
		-Oxygen sat.	-Body temp.		
		-BP	-Pupillary reaction		
		-Witnessed submersion	-Time of EMS assessment		
		-Time of EMS CPR	-CPR prior to EMS arrival		
		ED evaluation of vitals and aforementioned Info.			
		-Toxicology test	-Ventilation needs		
		-Serial neurological function			
		-Co-morbid illness -Other morbidities			
		-Outcome (death, neurological outcome)			
Weuster et al. [20]	N = 9	-Age	-Gender	-Duration of water emersion prior to submersion -Duration of submersion -Quality of CPR -Interval from rescue to CPR -Method of assessing neurological function/outcome -Premorbid functioning	Out of 9 participants: -4 were children. -All died due to cardiac arrest or cerebral hypoxia.
		-Body temp.	-GCS		
		-Body wt/ht	-BMI		
		-Outdoor temp.	-Water temp.		
		-Rescue Duration	-Preclinical CPR		
		-Duration of CPR	-ECLS/ECMO		
		-Cannulation	-Complications		
		-Weather	-Intubation		
		-CPR until ECMO/ECLS	-Mechanism of accident		
		-Inotropic/vasopressors	-Initial blood gases		
		-Neurological outcome (GCS/death)			
Suominen et al. [21]	N = 9	-Age	-Gender	-Duration of water emersion prior to submersion -Quality of CPR -Duration of CPR -If CPR was done prior to first responder arrival -Method of assessing neurological function/outcome -Premorbid functioning	Out of 9 children: -1 survived with mild to moderate neurologic deficit. -Slow rewarming with CPB may improve neurologic outcome. -The Pediatric Overall Performance Category Scale (POPC) was retrospectively used to assess neurological outcome at d/c and at one year follow-up.
		-Submersion time	-Water temp.		
		-Initial Rhythm	-Body temp. pH, BE		
		-Serum K+	-Duration of CPR		
		-1st responder call to arrival time			
		-Neurological Outcome			
